# Early life factors and being overweight at 4 years of age among children in Malmö, Sweden

**DOI:** 10.1186/1471-2458-10-764

**Published:** 2010-12-15

**Authors:** Elisabeth Mangrio, Martin Lindström, Maria Rosvall

**Affiliations:** 1 Department of Clinical Sciences, Social Medicine and Health Politics, Lund University, Malmö, Sweden; 2 Region Skåne, Malmö, Sweden

## Abstract

**Background:**

Rising rates of obesity and overweight is an increasing public health problem all over the world. Recent research has shown the importance of early life factors in the development of child overweight. However, to the best of our knowledge there are no studies investigating the potential synergistic effect of early life factors and presence of parental overweight on the development of child overweight.

**Methods:**

The study was population-based and cross-sectional. The study population consisted of children who visited the Child Health Care (CHC) centers in Malmö for their 4-year health check during 2003-2008 and whose parents answered a self-administered questionnaire (n = 9009 children).

**Results:**

The results showed that having overweight/obese parents was strongly associated with the child being overweight or obese. Furthermore, there was an association between unfavorable early life factors (i.e., mother smoking during pregnancy, presence of secondhand tobacco smoke early in life, high birth weight) and the development of child overweight/obesity at four years of age, while breastfeeding seemed to have a protective role. For example, maternal smoking during pregnancy was associated with an odds ratio (OR) of 1.47 (95% CI: 1.22, 1.76) for overweight and 2.31 (95% CI: 1.68, 3.17) for obesity. The results further showed synergistic effects between parental overweight and exposure to unfavourable early life factors in the development of child overweight.

**Conclusions:**

The present study shows the importance of early life factors in the development of child overweight and obesity, and thus puts focus on the importance of early targeted interventions.

## Background

Rising rates of obesity and overweight is an increasing public health problem all over the world [1-3]. In Sweden, about 15-20% of the children are overweight and 3-5% obese. During the past twenty years the prevalence of overweight children has doubled, while that of obese children has increased 4-5 times, even though the increase has started to level out during later years [4]. Throughout the world, overweight and obesity are occurring at continually younger ages [5] and children who are obese tend to be obese as adults [4].

This has vast implications for future public health, since it is well-known that obesity is associated with an increased risk of arteriosclerosis, pulmonary hypertension, ischemic heart disease, congestive heart failure [6], and asthma [7].

Obesity is a multifactorial disorder, and is the result of a joint effect of genetic and environmental factors. The most important environmental factors include the energy intake in relation to the need, and physical activity [8]. More recent research has also shown that an unfavourable environment early in life might elicit a range of physiological and cellular adaptive responses in key organ systems, which in turn might lead to pathology in later life [9]. Earlier studies have shown associations between early life factors such as maternal smoking during pregnancy [5,10-13], impaired fetal growth [9,10,14], low levels of breastfeeding [1,15], and high birth weight [10,16] in relation to overweight in children. The identification of risk factors in early childhood may allow early targeted interventions [12]. Furthermore, preschool age is considered as a critical period for obesity prevention [5]. Other factors of importance in the development of overweight and obesity among children are sociodemographic factors such as parental educational level [17] and origin of birth [18,19]. Genetic factors also seem to play an important role. Genome-wide association studies recently identified variants at several loci associated with body mass index [20]. Sweeting et al. [21] indicated that genetic factors determine individual susceptibility to weight gain, and several studies have shown an association between high parental BMI and overweight in children [1,22,23]. To the best of our knowledge, however, there are no studies that investigate the importance of early life factors in relation to presence or absence of parental overweight. This is an important topic since parental obesity might reflect adverse eating and physical activity patterns, and might be related to both early life factors and later obesity. Furthermore, genes shared between the parents and child might also be associated with both early life factors and later obesity [24]. The aim of this study was to investigate the association between early life factors and childhood overweight and obesity. We also wanted to investigate whether a potential association between early life factors and childhood overweight persisted after stratification for presence of parental overweight.

## Methods

### Study population

This study was conducted in Malmö, the third largest city in Sweden. According to statistics provided on the Malmö city website [25], the city has 293 909 inhabitants. It is a multiethnic city, with 171 different countries being represented. The study was population-based cross-sectional. The study population was 4 year old children from Malmö, who visited the Child Health Care (CHC) centres for their 4-year checkup during 2003-2008 and whose parents answered a self-administered questionnaire (n = 9, 009 children), i.e, 68% of the children who received the questionnaire. The CHC centers in Sweden is a well-established organization with the responsibility for reducing mortality, morbidity, and disability in newborn and younger children. Another purpose is to educate parents in making the most of their child's developmental opportunities. Through regular visits to the child health nurses, each child's weight and physical and developmental health are closely followed, and vaccinations are given until the child is 5-6 years old. The CHC focus is prevention; visits are voluntary and the consultations are free of charge. As many as 99% of children aged 0-6 participate in the program [26].

The data on the children in the present study was derived from a self-administered questionnaire distributed to parents of children who were registered with the CHC and invited for a 4-year checkup during the study period. The questionnaire was distributed by the pediatric nurses working at the CHC centers, and contained approximately 30 questions about the child's family situation, as well as the parents' education, occupation, country of birth, and financial and emotional security. It also included questions about exposure to maternal smoking during pregnancy, secondhand tobacco smoke, allergies, and breastfeeding. A pilot study was conducted where the questions were tested and validated at some of the CHC centers in Malmö before the questionnaire was taken into use [27]. The results of the pilot study showed that the questions generally were well understood with high response rates. However, there were some needs of corrections. For example, regarding environmental smoking, the words "also includes smoking outside" was added to the question and regarding the parents' educational level, the number of years of education was added. Furthermore, the results showed that there was a need for translating the questionnaire to other languages than Swedish and the questionnaire was after this translated into five different languages: Albanian, Arabic, English, Serbo-Croatian, and Somali [28]. In addition to the information provided by the parents, information was also collected from the CHC journal, i.e., the child's height and weight at 4 years, birth weight and whether the parents had taken part of the parental educational program. An earlier study showed only small differences between the participants and the non-participants with regard to parental place of birth (in Sweden vs. outside Sweden) and parental educational level [28]. The study was approved by the Regional Ethical Committee, Lund.

### Overweight

Children's height (cm) and weight (kg) was measured by the CHC nurse at the physical examination of the child at the CHC visit at 4 years. Overweight and obesity were assessed via iso-BMI according to the classification by Cole et al. based on six large nationally representative cross-sectional growth studies with cut-offs of 17.55 (boys) and 17.28 (girls) for overweight and 19.29 (boys) and 19.15 (girls) for obesity [29]. The parents' height and weight were self-reported in the questionnaire and overweight was defined as BMI > 25 kg/m^2 ^and obesity as BMI > 30 kg/m^2^.

### Sociodemographic factors and psychosocial factors

Maternal educational level was based on years of schooling and divided into low educational level (9 years or less), medium educational level (10-12 years), and high educational level (more than 12 years). Migration background was divided into three categories: both parents born outside Sweden, one parent born outside Sweden, and both parents born in Sweden. Position among siblings in the family was dichotomized into firstborn versus second born or later. Economic stress was assessed with the question "How many times during the past year did you not have enough money to afford the food or clothes that you and your family needed?", with answers being classified into yes (6 months a year or more) and no (very occasionally or never). Emotional support was assessed with the question "Do you have someone who can give you proper personal support to cope with life's stress and problems?", with answers being classified into low emotional support (not for certain or no) and high emotional support (definitely yes or probably yes). Practical support was assessed with the question "Would you be able to get help from someone to look after your children, within the same day?", with answers being classified into low practical support (not for certain or no) and high practical support (definitely yes or probably yes). Crowded living was defined as living in a household with more than two people per room (excluding the kitchen and bathroom). Parental training was assessed through CHC-journals where the nurse filled in whether the parents had taken part of the parental educational program or not.

### Early life factors

Maternal smoking during pregnancy was dichotomized into yes and no. *Secondhand tobacco smoke during early life *was assessed by the question: Did anyone in the family smoke when the child was 0-4 weeks of age? The answering alternatives were: No, yes - mother/stepmother smoked on a daily basis (also including outdoor smoking), or yes-father/stepfather smoked on a daily basis (also including outdoor smoking), or yes-siblings or other person smoked on a daily basis (also including outdoor smoking). *Secondhand smoking at 0-4 weeks of age *was divided into no (no secondhand tobacco smoke at all) and yes (daily secondhand tobacco smoke, including smoking outside). An identical question was used to assess *secondhand smoking at 8 months of age*. Birth weight was dichotomized into birth weight >4000 grams (high birth weight) and <=4000 grams (no high birth weight). Breastfeeding was classified into yes (1 month of breastfeeding or more) and no (0 months of breastfeeding).

### Child behavioural factors

The child's drinking of sweetened beverages (i.e., soft drinks, syrup or Coca-cola) was dichotomized into daily drinking of sweetened beverages versus no daily drinking of these beverages.

### Statistical methods

Logistic regression was used to analyze the associations between early life factors and presence of overweight/obesity at the age of 4 years. Multiple logistic regression analyses were performed in order to adjust the estimated odds ratios (OR) for the influence of potential confounding factors, i.e., sex, year, maternal educational level, parents' country of birth, participation in parental education program, first born, parental overweight, crowded living, intake of sweetened beverages and economic stress. Potential synergistic effects between presence of parental overweight and early life factors were tested using logistic regression analysis. A synergy index (SI) was calculated with the formula SI = (OR(AB) - 1)/((OR(Ab) - 1) + (OR(aB) - 1)), where OR = odds ratio, Ab = exposure to one risk factor, aB = exposure to the other risk factor and AB = exposure to both risk factors. Synergistic interaction was defined to be present if the effect of both exposures was more than additive compared with their independent effects (SI > 1) [30]. Confidence intervals (95%) for the synergy indexes were calculated [31]. Statistical analyses were performed with version 17.0 of SPSS for Windows.

## Results

In total, 1272 (15%) of the children were overweight and 264 (3%) were obese at 4 years of age. There were no statistically significant differences in prevalence between the different years, although there was a slight declining trend in overweight and obesity over time (data not shown).

Table 1 presents the prevalence of sociodemographic factors, parental overweight, lifestyle factors, early life factors, economic and psychosocial factors among normal weight, overweight and obese children. Overweight children were less often boys, more often had overweight parents, more often had a mother who smoked during pregnancy, more often had early exposure to secondhand tobacco smoke, more often had high birth weight and more often had parents who experienced economic stress compared to children who were normal weight. There were no statistically significant differences with regard to crowded living conditions, low parental educational level, parents born outside Sweden, no day care, parents having taken part in educational programs, being firstborn, intake of sweetened beverages, low social support, and being breastfed. A similar pattern of association was seen for presence of obesity at 4 years of age, but apart from the mentioned factors associated with overweight, obese children also more often had a mother or father with low education, had parents born outside Sweden, had parents who had not taken part of the parental educational program, lived in crowded conditions, and were less often breastfed compared to children who were not obese.

**Table 1:** Prevalence (%) of sociodemographic characteristics, life-style factors, early life factors, economic and psychosocial factors among normal-weight, overweight and obese 4-year old children in Malmö, Sweden.

	Child normal weight	Child overweight*†	Child obese†
	(n = 7349; 85%)	(n = 1272; 15%)	(n = 264; 3%)
*Sociodemographic factors*			
Male sex (%)	51.2	44.8‡	46.2‡
Both parents born outside Sweden (%)	28.7	28.8	34.2‡
Mother low education (%)	13.2	13.8	19.9‡
Father low education (%)	14.3	15.6	22.1‡
Firstborn (%)	46.1	44.8	41.9
No day care (%)	4.6	4.1	7.5
Crowded living (%)	10.8	11.1	15.5‡
Not taken part of parental education (%)	47.4	49.7	54.4‡
*Parental overweight*			
Mother overweight (%)	27.3	37.6‡	49.3‡
Father overweight (%)	52.1	69.3‡	73.7‡
Both parents overweight (%)	17.0	29.1‡	40.0‡
*Life style factors*			
Child's intake of sweetened beverages (%)	10.5	12.0	12.7
Secondhand tobacco smoke at 4 years of age (%)	22.6	26.4‡	32.6‡
*Early life factors*			
Mother smoking during pregnancy (%)	9.6	13.6‡	20.0‡
Secondhand tobacco smoke at 0-4 weeks (%)	22.3	26.1‡	33.6‡
Secondhand tobacco smoke at 8 months (%)	24.8	28.2‡	37.0‡
High birth weight (>4000 g) (%)	15.8	24.0‡	25.6‡
Not breastfed (%)	3.9	5.0	6.5‡
*Economic and psychosocial factors (%)*			
Parental low emotional support (%)	21.1	22.3	26.3
Parental low practical support (%)	29.8	29.5	28.9
Parental economic stress (%)	6.1	8.0‡	9.6‡

* Also including obese children.

† Child overweight was defined as iso-BMI > 17.55 (boys) and 17.28 (girls); child obesity as iso-BMI > 19.29 (boys) and 19.15 (girls). ‡ Statistically significantly (p < 0.05) different compared to normal-weight children, after adjustment for year and sex.

In total 34% of the 4-year old children had normal-weight parents, while 66% had at least one overweight parent. Table 2 shows the odds ratios of overweight and obesity among 4-year-old children in Malmö, Sweden in relation to early life factors. In model 1, adjustments were made for year and sex. In model 2, adjustments were made for year, sex, maternal educational level, parents' country of birth, crowded living, economic stress, being firstborn, and participation in a parental educational program. In model 3 additional adjustments were made for parental overweight and intake of sweetened beverages. Maternal smoking during pregnancy increased the odds for both overweight and obesity and remained statistically significant in the adjusted models. Presence of secondhand tobacco smoke at the age of 0-4 weeks and 8 months also showed associations with overweight and obesity, respectively, which remained statistically significant after adjustment for potential confounders. Having a high birth weight was associated with increased odds of overweight and obesity, but turned statistically non-significant in the last model with regard to obesity. Not being breastfed was associated with increased odds of obesity and tended to be associated with increased odds of overweight, however, it turned non-significant after additional adjustment for potential confounders.

**Table 2:** Odds ratios (95% confidence intervals) of overweight and obesity in 4-year old children in Malmö, Sweden, by early life factors.

	Overweight*			Obesity*		
	
	OR (95% CI)*	OR (95% CI)	OR (95% CI)	OR (95% CI)*	OR (95% CI)	OR (95% CI)
	Model 1†	Model 2‡	Model 3‡‡	Model 1†	Model 2‡	Model 3‡‡
***Early life factors***						
*Mother smoking during pregnancy*						
Yes	1.47 (1.22, 1.76)	1.43 (1.16, 1.76)	1.48 (1.17, 1.87)	2.31 (1.68, 3.17)	2.07 (1.42, 3.03)	2.10 (1.37, 3.23)
No	1.00	1.00	1.00	1.00	1.00	1.00
*Secondhand tobacco smoke (0-4 weeks)*						
Yes	1.23 (1.07, 1.42)	1.32 (1.12, 1.54)	1.35 (1.13, 1.61)	1.75 (1.34, 2.27)	1.62 (1.19, 2.21)	1.54 (1.08, 2.19)
No	1.00	1.00	1.00	1.00	1.00	1.00
*Secondhand tobacco smoke (8 months)*						
Yes	1.19 (1.03, 1.36)	1.26 (1.07, 1.48)	1.30 (1.09, 1.57)	1.76 (1.35, 2.29)	1.61 (1.17, 2.20)	1.51 (1.06, 2.16)
No	1.00	1.00	1.00	1.00	1.00	1.00
*High birth weight (>4000 g) (%)*						
Yes	1.75 (1.51, 2.02)	1.70 (1.44, 2.00)	1.51 (1.26, 1.81)	1.91 (1.43, 2.55)	1.81 (1.29, 2.53)	1.47 (0.99, 2.16)
No	1.00	1.00	1.00	1.00	1.00	1.00
*Breastfed (%)*						
No	1.30 (0.97, 1.74)	1.27 (0.91, 1.76)	1.37 (0.96, 1.96)	1.76 (1.04, 2.96)	1.60 (0.87, 2.93)	1.68 (0.86, 3.29)
Yes	1.00	1.00	1.00	1.00	1.00	1.00

* OR, odds ratio; CI, confidence interval; overweight, including overweight and obesity (i.e., iso-BMI > 17.55 (boys) and 17.28 (girls)); obesity, obese children (i.e., iso-BMI > 19.29 (boys) and 19.15 (girls)) compared to normal weighted children.

† Adjusted for year and sex.

‡ Adjusted for year, sex, maternal educational level, parents' country of birth, crowded living, being firstborn, having taken part pf parental educational program, and economic stress.

‡‡ Model 2 with additional adjustment for parental overweight and child's intake of sweetened beverages.

Table 3 presents presence of sociodemographic factors, life style factors, early life factors, economic and psychosocial factors in four groups constructed based on presence of child overweight and presence of parental overweight. The four groups were: normal-weight children with normal-weight parents, overweight children with normal-weight parents, normal-weight children with at least one parent being overweight and overweight children with at least one parent being overweight. The table shows that compared to normal-weight children with normal-weight parents, children with at least one overweight parent generally more often had a mother with low educational level, had parents born outside Sweden, had parents who did not participate in parental educational programs, had parents with low emotional support, and had parents who experienced economic stress. Furthermore, these children had more unfavorable early life factors, more often lived in crowded conditions, were less often firstborn, and more often had a daily intake of sweetened beverages. Among the children with overweight parents, overweight children were less often boys and generally had more unfavorable early life factors than normal-weight children, i.e., these children more often had a mother who smoked during pregnancy, had presence of secondhand tobacco smoke during their first month in life, had a high birth weight, and were less often breastfed compared to normal-weight children with overweight parents.

**Table 3:** Means and prevalences (%) of sociodemographic characteristics, life-style factors, early life factors, and economic and psychosocial factors, by presence of parental and child overweight in 4-year old children in Malmö, Sweden.

	Both parents normal-weight		At least one parent overweight*	
	Child overweight†		Child overweight†	
	No	Yes	No	Yes
*Sociodemographic factors*				
Male sex (%)	50.8	44.7	52.3	44.7‡§
Both parents born outside Sweden (%)	19.2	15.7	31.3‡	29.5‡
Mother low education (%)	7.4	7.1	13.5‡	12.5‡
Firstborn (%)	50.6	46.7	43.8‡	45.1‡
No day care (%)	3.8	4.6	4.7	3.6
Mother's weight	60.2	61.0	71.4‡	73.8‡
Father's weight	74.3	75.9	89.6‡	89.9‡
Crowded living (%)	6.7	7.9	11.8‡	10.5‡
Not taken part of parental education (%)	42.7	48.4	48.0‡	50.2‡
*Life style factors*				
Child having a daily intake of sweetened beverages (%)	7.9	8.3	12.0‡	12.7‡
Secondhand tobacco smoke at 4 years of age (%)	16.5	24.4‡	23.9‡	25.9‡
*Early life factors*				
Mother smoking during pregnancy (%)	6.7	5.7	10.2‡	15.5‡§
Secondhand tobacco smoke at 0-4 weeks (%)	16.2	21.2	23.4‡	27.2‡§
Secondhand tobacco smoke at 8 months (%)	18.5	22.3	26.2‡	29.7‡
High birth weight (%)	15.0	17.6	17.2‡	25.5‡§
Not breastfed (%)	2.7	3.2	4.3‡	6.1‡§
*Economic and psychosocial factors (%)*				
Parental low emotional support (%)	16.5	15.9	21.4‡	22.5‡
Parental low practical support (%)	29.0	27.1	28.3	28.9
Parental economic stress (%)	4.2	5.1	6.2‡	8.1‡

* Parental overweight was defined as having a BMI > 25 kg/m ^2.^

† Child overweight was defined as iso-BMI > 17.55 (boys) and 17.28 (girls).

‡ Statistically significantly (p < 0.05) different compared to normal-weight children with normal-weight parents after adjustment for year and sex.

§ Statistically significantly (p < 0.05) different compared to normal-weight children with at least one overweight parent after adjustment for year and sex.

Table 4 presents the odds ratios of child overweight at 4 years of age in relation to presence of maternal smoking during pregnancy stratified by presence of parental overweight. Children whose mothers had not smoked during pregnancy and with normal-weight parents were used as the reference group. Children with presence of maternal smoking during pregnancy and with at least one overweight parent showed highly increased odds of being overweight at 4 years of age, while no such effect was seen among children with presence of maternal smoking during pregnancy whose parents were normal-weight. The synergy index was 3.12 (95% CI: 1.13, 8.63). As this value is larger than 1, it indicates a synergistic effect of the mother's smoking during pregnancy and having overweight parents on the child being overweight at the age of 4 years. Additional adjustment for confounders did not change this association (table 4). A similar pattern of association was seen for child obesity with corresponding ORs of 0.51 (95% CI: 0.07, 3.87), 2.82 (95% CI: 1.86, 4.28) and 6.87 (95% CI: 4.13, 11.42). The synergy index was 4.41 (95% CI: 1.55, 12.54). Moreover, a similar pattern of association was observed after stratification for parental obesity instead of overweight with a synergy index of 4.95 (95% CI: 2.03, 12.04) for child obesity.

**Table 4:** Odds ratios (OR) and 95% confidence intervals (CI) of being overweight at 4-years of age by maternal smoking during pregnancy and presence of parental overweight, Malmö, Sweden.

	Parents normal-weight		At least one parent with an overweight*	
		
	Maternal smoking during pregnancy		Maternal smoking during pregnancy	
		
	No	Yes	No	Yes
	OR; 95% CI*	OR; 95% CI	OR; 95% CI	OR; 95% CI
*Adjusted for sex and year*	1.00†	0.84 (0.47, 1.50)	1.73 (1.47, 2.04)	2.77 (2.16, 3.55)§
*Adjusted model‡*	1.00†	0.98 (0.53, 1.92)	1.75 (1.46, 2.10)	2.81 (2.11, 3.73)§

* OR, odds ratio; CI, confidence interval; Parental overweight was defined as having a BMI > 25 kg/m^2 ^† Reference level.

‡ Adjusted for year, sex, maternal educational level, parents' country of birth, crowded living, being firstborn, having taken part pf parental educational program, economic stress, and intake of sweetened beverages.

§ Statistically significantly different (p < 0.05) from the category: At least one parent with an overweight and no maternal smoking during pregnancy.

Table 5 presents the odds ratios of child overweight at 4 years of age in relation to presence of high birth weight stratified by presence of parental overweight. Children with no high birth weight and with normal-weight parents were used as the reference group. Children with high birth weight and with at least one overweight parent showed highly increased odds of being overweight at 4 years of age, while no such effect was seen among children with high birth weight whose parents were normal-weight. The synergy index was 2.00 (95% CI: 1.15, 3.47). As this value is larger than 1, it indicates a synergistic effect between high birth weight and having overweight parents. Additional adjustment for confounders did not change this association (table 5). A similar pattern of association was seen for obesity with corresponding ORs of 0.45 (95% CI: 0.07, 3.87), 2.67 (95% CI: 1.74, 4.10) and 4.84 (95% CI: 2.96, 7.92). The synergy index was 3.45 (95% CI: 1.30, 9.16). Similar, but weaker, patterns of associations were also seen for not having been breastfed and presence of secondhand tobacco smoke at 0 to 4 weeks and at 8 months, respectively (data not shown). Moreover, stratification for parental obesity instead of overweight generally showed similar synergy indexes as stratification by parental overweight.

**Table 5:** Odds ratios (OR) and 95% confidence intervals (CI) of being overweight at 4-years of age by presence of high birth weight and presence of parental overweight, Malmö, Sweden.

	Parents normal-weight		At least one parent with an overweight*	
		
	High birth weight (>4000 g)		High birth weight (>4000 g)	
		
	No	Yes	No	Yes
	OR; 95% CI*	OR; 95% CI	OR; 95% CI	OR; 95% CI
*Adjusted for sex and year*	1.00†	1.27 (0.88, 1.83)	1.73 (1.45, 2.06)	2.96 (2.37, 3.69)§
*Adjusted model‡*	1.00†	1.26 (0.86, 1.86)	1.74 (1.44, 2.11)	2.76 (2.17, 3.52)§

* OR, odds ratio; CI, confidence interval; parental overweight was defined as having a BMI > 25 kg/m^2. ^† Reference level.

‡ Adjusted for year, sex, maternal educational level, parents' country of birth, crowded living, being firstborn, having taken part of parental educational program, economic stress, and intake of sweetened beverages.

§ Statistically significantly different (p < 0.05) from the category: At least one parent with an overweight and no high birth weight.

## Discussion

The results showed that having overweight/obese parents was strongly associated with the child being overweight or obese. Furthermore, there was an association between unfavorable early life factors (mother smoking during pregnancy, presence of secondhand tobacco smoke early in life, high birth weight) and the development of child overweight/obesity at 4 years of age, while breastfeeding seemed to have a protective role. After stratification for parental overweight, such associations were only found in children whose parents were overweight/obese and not among children with normal-weight parents. This indicates a synergistic effect between presence of parental overweight and unfavorable early life factors.

Having overweight/obese parents was strongly associated with the child being overweight or obese. This result is in line with the results from other studies [1,22,23]. This association could be due to genetic factors, as well as environmental and/or behavioral factors. According to Crocker et al. [32], the environment around the child seems to play an important role in determining whether he or she becomes obese. This observation is demonstrated by comparing human samples that share genetic background but are raised in different cultures and have a different outcome regarding obesity. Scaglioni et al. [33] reports that parents play an important role in development of their children's food preferences and energy intake. Research also indicates that feeding practices, such as exerting excessive control over what and how much the child eats, can contribute to the child's becoming overweight.

Early life has been seen as a critical period for the development of obesity. Early life factors such as environmental smoking [1,10,11], maternal smoking [5,10-13], impaired fetal growth [9,10], high birth weight [10,16] and low levels of breast feeding [1,15] have previously been shown to be associated with child overweight. In a review by Gluckman and Hanson published in 2004, it was concluded that early development make important echoes in disease risk throughout life, and that this is important to bear in mind when forming interventional strategies [34]. An unfavourable environment in early life is thought to elicit a range of physiological and cellular adaptive responses in key organ systems. These adaptive changes result in permanent alterations and might lead to pathology in later life. However, the mechanisms underlying the developmental origins of disease remain poorly defined [9]. Environmentally induced changes in epigenetic states could lead to dysfunctional organ growth and differentiation that is detrimental in the long term [9]. There are theories involving an altered glucose-insulin metabolism in utero leading to an increased risk for obesity later in life. Many studies have shown an association between high birth weight and increased childhood as well as adult BMI [16,35-38]. Most of the studies on potential mechanisms between high birth weight and later adiposity come from studies of diabetes during pregnancy [24]. Maternal hyperglycemia leads to excess fetal insulin, which in turn acts as a growth hormone for the fetus. Furthermore, animal studies suggest that fetal hyperinsulinemia can alter expression of hypothalamic neurotransmitters, leading to offspring hyperphagia and increased weight [37]. Breastfeeding has been shown to protect against obesity in childhood and in adolescence [15,39,40]. One mechanism is the related to the higher protein/nitrogen content of infant formula compared with breast milk, which may cause increased insulin and insulin-like growth factor-1 secretion leading to excessive weight gain [15]. In addition, according to Balaban and Silva [41], the mechanism behind lack of breastfeeding and obesity might be related to metabolic imprinting, which causes the early nutritional experience to result in long lasting effects that predisposes to certain diseases. Long-term effects of maternal smoking in pregnancy on the risk of overweight might be related to long-term effects of nicotine exposure on neurobehavioural impulse control affecting satisfaction and appetite leading to an increased food consumption [42] as well as through poor nutrition in the uterus [43,44]. The process which link reduced fetal growth with overweight/obesity has been suggested to stem from adrenal overactivity initiated by early growth restraint [45] and by early postnatal catch-up growth resulting in an acceleration of growth postnatally that overshoots the genetic trajectory [43]. Secondhand tobacco smoke could be related to overweight/obesity via low parental education and less healthy food patterns [46]. However, the association in the present study between secondhand tobacco smoke and overweight persisted even after adjustment for the mother's educational level.

In the present study, exposure to unfavorable early life factors and having overweight parents seemed to have a synergistic effect on the development of child overweight or obesity. Rothman's model of synergism used in the present study is based on the theory of two causes being component causes in the same sufficient cause [47]. The criterion for interaction is departure from additivity and the reference group for the comparisons is the group unexposed to either factor. To the best of our knowledge there are no studies that specifically investigate the importance of early life factors in relation to presence or absence of parental overweight.

Doing so is important since parental obesity might reflect adverse eating and physical activity patterns, and might be related to both early life factors and later obesity. Furthermore, genes shared between the parents and child is associated with early life factors such as birth weight and later obesity [24]. Since the first years in life constitute a critical period for the development of overweight, it is important to study children of preschool age and to focus on the importance of early targeted interventions [5,12].

Certain methodological issues need to be addressed. Parents with 4-year-old children were invited to participate in the study by answering the questionnaire. The total number of children whose parents answered the questionnaire during the study period was 9009, or about two thirds of all who received the questionnaire. An earlier study showed only small differences with regard to country of birth and educational level between the participants and non-participants [28]. One strength of this study was the fact that the questionnaire had been tested for validity and translated into five different languages [28]. These languages are among the most common languages represented in Malmö [25]. However, there may still have been some bias, as despite the multiple translations some parents may have had difficulties in understanding the questions. Another problem with the questionnaire was that it contained only one question on nutrition. It would have been interesting to have more information about the food eaten by the children. In our study, there were only small differences in intake of sweetened beverages between normal-weight and overweight children, even though there has been increasing awareness of the contributing role these beverages play in the development of obesity in children [23]. Similar results were found in another study from the United States where increased beverage consumption was associated with an increase in the total energy intake of preschool children, but not with their BMI [14]. According to Kleiser [10], it is not easy to measure nutrition and physical activity in large scale studies, as the instruments used for measuring are too crude to allow the drawing of conclusions. We chose to adjust for the following confounding factors: year, sex, maternal education level, parents' origin of birth, crowded living conditions, being firstborn, parental overweight, having taken part of parental educational programs, economic stress, and intake of sweetened beverages. However, there might have been other confounding factors not included in our models.

## Conclusion

Rising rates of obesity and overweight is an increasing public health problem all over the world. The present study shows the importance of early life factors in the development of child overweight and obesity, and thus puts focus on the importance of early targeted interventions. The results further showed a synergistic effect of parental overweight and exposure to unfavorable early life factors on the development of child overweight and obesity.

## Competing interests

The authors declare that they have no competing interests.

## Authors' contributions

EM and MR have contributed to the conception of the work, the analysis of the data, the interpretation and the discussion of the results, the drafting, writing and revision of the content. ML has contributed to the drafting and revision of the content. All authors have read and approved the final manuscript.

## Pre-publication history

The pre-publication history for this paper can be accessed here:

http://www.biomedcentral.com/1471-2458/10/764/prepub
